# Aducanumab: a new phase in therapeutic development for Alzheimer’s disease?

**DOI:** 10.15252/emmm.202114781

**Published:** 2021-08-02

**Authors:** Giovanna Lalli, Jonathan M Schott, John Hardy, Bart De Strooper

**Affiliations:** ^1^ UK Dementia Research Institute at UCL London UK; ^2^ Dementia Research Centre UCL Queen Square Institute of Neurology London UK; ^3^ UK Dementia Research Institute at UCL London UK; ^4^ Reta Lila Weston Institute UCL Queen Square Institute of Neurology London UK; ^5^ Department of Neurodegenerative Disease UCL Queen Square Institute of Neurology London UK; ^6^ Institute for Advanced Study The Hong Kong University of Science and Technology Hong Kong China; ^7^ Department of Neurosciences Leuven Brain Institute VIB Centre for Brain & Disease Research and KU Leuven Leuven Belgium

**Keywords:** Neuroscience

## Abstract

On 7 June_,_ the FDA approved aducanumab, the first new drug for Alzheimer’s disease in almost 20 years—and notably, the first drug with a putative disease‐modifying mechanism for the treatment of this devastating disorder, namely the removal of β‐amyloid (or Aβ) plaques from the brain.

## Graphical abstract

G. Lalli, J. Schott, J. Hardy, and B De Strooper comment on the FDA approval of aducanumab and provide some interesting points to consider.

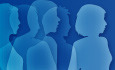

The approval of aducanumab has proven to be highly controversial and has sparked global debate, with contrasting opinions dividing the scientific community (see, for example, Mullard, 2021 and Perlmutter, 2021). The results from two phase III randomized clinical trials with aducanumab were far from conclusive, with conflicting evidence for clinical improvements. These, where present, were of questionable clinical relevance (Liu *et al*, 2021). Instead, the FDA noted the consistent and convincing reduction in brain β‐amyloid plaques (measured by PET scanning) in a dose‐ and time‐dependent fashion with some evidence for alterations in presumed downstream biomarkers, concluding that “the reduction in plaques is reasonably likely to result in clinical benefit” (https://www.fda.gov/drugs/news‐events‐human‐drugs/fdas‐decision‐approve‐new‐treatment‐alzheimers‐disease). The FDA granted Accelerated Approval based on the clear effect on the biomarker, the possible clinical effect seen in one trial and the urgent need in this area of medicine. The FDA required a phase IV post‐marketing randomized controlled trial to verify clinical benefit with submission of final report expected by February 2030, which seems to us rather late.

Prior to this decision, the history of drug development in Alzheimer’s had largely been discouraging, including more than 25 negative randomized clinical trials testing the “amyloid cascade hypothesis” (Alexander *et al*, 2021). However, these failures have not deterred continued efforts targeting Aβ in different biophysical states, i.e. monomer, oligomer, amyloid fibrils and amyloid plaques (Cummings *et al*, 2019); and indeed, promising results from phase II clinical trials with other anti‐Aβ monoclonal antibodies, including gantenerumab (Klein *et al*, 2019), lecanemab (Swanson *et al*, 2021) and more recently donanemab (Mintun *et al*, 2021) have led to ongoing phase III studies. It is notable that the decision to license aducanumab on the basis of amyloid removal has rapidly led to lecanemab and donanemab to be granted “Breakthrough Therapy” designation by the FDA in June 2020, with submission for accelerated approval coming up in the near future (http://www.pharmatimes.com/news/lecanemab_wins_breakthrough_therapy_designation_in_alzheimers_disease_1372104; https://investor.lilly.com/news‐releases/news‐release‐details/lillys‐donanemab‐receives‐us‐fdas‐breakthrough‐therapy).

Amyloid plaques are mainly formed by amyloid peptides of 36‐43 amino acids deriving from cleavage of amyloid precursor protein (APP) by beta‐ and gamma‐secretases. Mutations in APP or gamma‐secretases cause rare forms of dominantly inherited Alzheimer’s disease. The question of the role of amyloid plaques in Alzheimer’s disease is complex. The way the field has approached their role, however, has often been simplistic, with individuals taking binary sides either “for” or “against” the amyloid hypothesis. Our take is more nuanced: there is convincing genetic and basic research to suggest that amyloid peptides are early and necessary for the development of Alzheimer’s disease and are not passive bystanders. On the other hand, there is also compelling evidence that a simplistic cause–consequence relationship between accumulating amyloid and neurodegeneration should have been abandoned more than a decade ago (Karran *et al*, 2011). Previous work has proposed that amyloid pathology acts as a trigger for a series of cellular processes that evolve over time and lead, only relatively late, to neurodegeneration and dementia (De Strooper & Karran, 2016). Crucial questions regarding what a minimal threshold of amyloid pathology is and whether these cellular disease processes evolve at a certain moment independently of amyloid pathology remain unanswered. Similarly, the question remains whether amyloid removal on its own will provide clinical benefits in patients suffering from mixed forms of dementia (amyloid plaques and tangles are frequently associated with vascular pathologies, alpha‐synuclein or TDP‐43 inclusions, and other alterations). Most importantly, there is good evidence that amyloid pathology accumulates for more than a decade before clinical manifestations, suggesting that the timing of drug administration during this process is also important. It is not clear whether removal of amyloid will stop downstream processes that have already started; even if that should be the case, it may take some time to remove the accumulated amyloid from the brain to reach a level below the pathological threshold, and it is unclear whether repeated dosing will be needed to maintain this. It is also unclear whether any clinical benefits that might be seen are due to the halting of downstream neurodegeneration or, if caught early enough, whether any restoration of brain function is possible. With aducanumab, we can now start to address some of those questions.

Importantly, Alzheimer’s disease is a complex neurodegenerative disorder (Scheltens *et al*, 2021), and apart from the anti‐amyloid therapies, there are numerous drugs in the pipeline, which target other hallmarks of the disease such as Tau‐tangles, synaptic failure, neuroinflammation and vascular pathology (Cummings *et al*, 2021). The fact that at the time of writing two other antibodies targeting amyloid are on the same accelerated approval track as aducanumab may have advantages, driving down prices and increasing the urgency of clinical trials to show efficacy, but it is important that the current focus on amyloid immunotherapy does not lower efforts in other areas of drug discovery, as the Alzheimer’s drug development pipeline extends well beyond β‐amyloid (Cummings *et al*, 2021).

In the clinical trials, aducanumab was associated with amyloid‐related imaging abnormalities (ARIA)—either brain oedema (ARIA‐E) or new microhaemorrhages/siderosis (ARIA‐H)—observed in 41% of individuals treated with the highest dose (10 mg/kg) compared with 10% on placebo. While most of these abnormalities were asymptomatic, and of the 24% who did develop treatment‐related symptomatic ARIA the majority resolved spontaneously, occasionally patients developed serious symptoms. While the prescribing indications for aducanumab mandate routine MRI screening for asymptomatic ARIA and provide guidance for how symptomatic cases should be managed, the side effect profile of aducanumab in a “real‐life” scenario, e.g. in patients with mixed or more advanced disease or in less specialist settings, is yet to be established. It is not yet clear whether this will impact on risk/benefit calculations and the overall costs of administering treatment.

Another important ethical consideration is raised by the very high price for this medication set at about $56,000 per year per person (Mullard, 2021). While the drug clearly removes fibrillar amyloid from the brain, and while it is not unreasonable to suppose that this might ultimately result in some clinical benefit at least in a subset of the patients, the current evidence supporting meaningful clinical benefit is, at best, thin (e.g. with effects on MMSE change from baseline smaller than those observed with the acetylcholinesterase inhibitor donepezil—Liu *et al*, 2021). The current level of evidence for efficacy, the uncertainty about the length of treatment and the high costs—higher still when the costs of screening and safety surveillance are included—inevitably lead to question cost/benefit and who will pay the bill. Hope has been raised with desperate patients, many of whom will request access to aducanumab and will be ready to pay what they can. There will be expectations from many that health insurance companies and governments will help those who cannot afford the price. And, even if the drug is ultimately proven to be effective and funds made available, there are major challenges to ensure widespread delivery, as no healthcare system is yet ready to accommodate the demand across all the patients who might benefit. More discussion is needed to see how society can move forward in a wise and sustainable way with this and other novel medications that will hopefully be available for dementia before long.

One concern is that the approval of aducanumab (at least in the United States) will either lead to nihilism in the sceptical, or to widespread prescription across a range of disease severity amongst enthusiasts. The field has now a tool to evaluate the usefulness of an anti‐amyloid therapeutic that has a demonstrated effect on one of the hallmarks of Alzheimer’s disease. We can now address the questions mentioned above in well‐designed clinical trials. Which patients will have benefit most from this drug? At what disease stage maximal cost/benefit can be achieved? While the licence for aducanumab appears to allow for treatment across the severity of Alzheimer’s disease (see Note added in proof), it seems likely that maximum benefits—if any—will be seen when given early. And the ultimate test to definitively determine the triggering role of amyloid plaques in the cellular processes that eventually lead to Alzheimer’s disease will be to test carefully selected patients with positive biomarkers but no clinical symptoms (including individuals with familial Alzheimer’s disease—Salloway *et al*, 2021) who should be followed up over years including through a wide range of biomarkers to see whether early intervention with aducanumab—or in due course with other amyloid‐removing therapies—does halt Alzheimer’s disease in its tracks.

At the time of writing, the design of the post‐approval phase IV trial requested by the FDA is unknown. It is vital that this/these trial(s) are designed carefully in collaboration with the academic community and address specific aspects of the remaining questions in a refined and conclusive way by selecting patients, biomarkers and cognitive outcomes in a logical and consistent manner. Hard proof of efficacy should be sought in well‐defined Alzheimer’s disease cases, which, of note, are relatively rare in the population. Aducanumab might turn out to also be useful in later cases or in patients with mixed dementia, but it would be unwise to diminish the chance of showing beneficial effects by including patients in whom there are good theoretical grounds for expecting limited or no effects, and indeed more side effects, at this moment.

In parallel, for the field to move forward and learn important lessons that may change the future of clinical trials in Alzheimer’s disease, it will be crucial that Biogen and its partners release all available clinical trial data to the community in a form that allows re‐analysis. This would increase confidence and accelerate progress in better understanding which patient groups may benefit from treatment, besides encouraging the wider community to develop innovative trial designs for future therapies.

Whatever one’s views of the FDA’s decision, the accelerated approval of aducanumab demonstrates that the FDA now deems Alzheimer’s disease to be a sufficiently important unmet need to warrant approval based on biomarker changes. This is akin to the early times in AIDS research when drugs with minimal clinical benefit were fast tracked for use in humans because of an effect on a surrogate endpoint. Indeed, it was the AIDS pandemic that led the FDA to innovatively streamline the approval process for promising new antiretroviral agents and later for molecular diagnostics (Broder, 2010). This was instrumental for opening the way to further clinical experimentation and trialling, crucially attracting long‐term investment of pharma/biotech. While it is true that drugs reached the clinic with modest clinical benefit, this more dynamic and risk‐taking approach has brought the AIDS field within two decades to almost curative therapies. Time will tell, but we may now be at a similar pivotal moment for Alzheimer’s field—where a critical approval decision could spark a transformative reinvigoration of efforts in research and drug development.

In conclusion, progress in defeating Alzheimer’s disease is not helped by polarized yes–no discussions which have paralyzed our field already many times in the past. Alzheimer’s disease and other forms of dementia are complex disorders, which commonly coexist, and akin to cancer, it is not feasible that one simple hypothesis and one silver bullet will explain and treat all. In the past, many trials failed to consistently and effectively remove β‐amyloid or were stopped because of side effects more severe than those linked to aducanumab. The approval of aducanumab—at least in the USA—is now a reality, and, whatever one’s views about the decision, it provides an unprecedented opportunity to learn about Alzheimer’s disease pathogenesis and hopefully lead to novel ways to treat dementia. One big lesson is already that interim futility analyses (which led to the aducanumab trials being aborted prematurely and to all subsequent decisions being made on incomplete data) have significant limitations. The use of the accelerated approval pathway has confirmed the vital role of biomarkers in clinical trials of dementia as both inclusion and outcome measures and paves the way for new treatments to move faster into the clinic. There has been speculation that the controversy and costs of aducanumab will stifle research, investment and innovation in Alzheimer’s disease. This was not seen in other fields where such decisions were taken. Despite early approval of drugs with weak clinical benefits for AIDS, multiple sclerosis or cancer, further therapeutic developments went on. Once it became clear for industry that a clear path to the market is there, an explosion of novel drugs and clinical experimentation followed. This, eventually, led to dramatic improvements in clinical outcomes in these fields. We believe that the approval of aducanumab and the pending approvals of other anti‐amyloid therapies will open a new phase of intense basic and clinical research into Alzheimer’s disease, which will ultimately bring light at the end of a long dark tunnel of past disappointments and false dawns. While the problematic aspects of the aducanumab approval have been debated at length and, importantly, efficacy has not yet been conclusively established, we hope this decision will ultimately be seen as the first (small) step towards disease‐modifying therapies for Alzheimer’s disease.

## Conflict of interest

GL has no competing interests. JMS has consulted for Biogen and was a member of the EMERGE and ENGAGE Interpretation Task Force. He is Chief Medical Officer of Alzheimer’s Research UK and Medical Advisor to the UK Dementia Research Institute. Views expressed here do not necessarily reflect those of these organizations. JH is an occasional consultant for Eisai on their microglial programme and an advisor for Eli Lilly on their amyloid programme. BDS is consultant for Eisai, ReMYND NV, AbbVie and Muna‐K5. He is the scientific founder of Augustine TX and Muna‐K5 and has a small amount of shares from Muna‐K5. He is director of the UK Dementia Research Institute. The commentary reflects his personal views. BDS is a senior academic editor of *EMBO Molecular Medicine*.

## Note added in proof

After acceptance of this article for publication, the FDA modified its guidance on aducanumab on 8 July 2021 to narrow the patient spectrum to mild Alzheimer's disease and Mild Cognitive Impairment (MCI) (biogencdn.com/us/aduhelm‐pi.pdf).
